# Supplementary material for: Reproductive ecology of the endangered Beal’s-eyed turtle, Sacalia bealei
Source: PeerJ. 2018 Jun 14;6:e4997. doi: 10.7717/peerj.4997 (PMC6018645; doi:10.7717/peerj.4997)
Supplement: Supplemental Information 1 [file peerj-06-4997-s001.docx]

Table1 Distance of nests from the stream

| Turtle ID | Straight Distance from nest to stream bank (m) | Straight Distance from nest to site emerging from water (m) | Straight Distance from nest to site returning to water (m) | Whole crawling distance during nest selection (m) |
| --- | --- | --- | --- | --- |
| 13 | 7.00 | 9.00 | 13.00 | 30.50 |
| 14 | 7.80 | 11.50 | 18.00 | 38.00 |
| 15 | 11.00 | 13.00 | 17.00 | 67.00 |
| 17 | 13.00 | 17.00 | 20.50 | 47.50 |
| 18 | 8.00 | 10.00 | 26.00 | 55.00 |
| 20 | 4.50 | 8.30 | 4.80 | 18.00 |
| Mean±SE | 8.55±1.23 | 11.47±1.31 | 16.55±2.93 | 42.67±7.18 |
