# Supplementary material for: Reproductive ecology of the endangered Beal’s-eyed turtle, Sacalia bealei
Source: PeerJ. 2018 Jun 14;6:e4997. doi: 10.7717/peerj.4997 (PMC6018645; doi:10.7717/peerj.4997)
Supplement: Supplemental Information 2 [file peerj-06-4997-s002.docx]

Table 2 Characters of nesting female *S. bealei* and their fecundity

| Turtle ID | Body Weight (g) | Carapace Length (mm) | Carapace Width (mm) | Plastron Length (mm) | Plastron Width (mm) | Body Height (mm) | Clutch size | No. of Fertilized Eggs | Egg length (mm) | Egg width (mm) | Egg weight (g) | RCM (%) | REM (%) |
| --- | --- | --- | --- | --- | --- | --- | --- | --- | --- | --- | --- | --- | --- |
| 13 | 340 | 133.9 | 94.6 | 121.2 | 78.6 | 50.3 | 3 | 2 | 42.1 | 24 | 14 | 11.47 | 3.82 |
|  |  |  |  |  |  |  |  |  | 46 | 23.6 | 12 |  |  |
|  |  |  |  |  |  |  |  |  | 46.7 | 24.5 | 13 |  |  |
| 14 | 378 | 143 | 96.8 | 126.9 | 77.3 | 53.9 | 2 | 2 | 49.4 | 25 | 18.7 | 9.76 | 4.89 |
|  |  |  |  |  |  |  |  |  | 49.5 | 24.9 | 18.2 |  |  |
| 15 | 298 | 128.3 | 93.3 | 117.8 | 77.3 | 57.1 | 2 | 2 | 49.5 | 24.9 | 18.2 | 12.21 | 6.11 |
|  |  |  |  |  |  |  |  |  | 46.5 | 26.1 | 18.2 |  |  |
| 17 | 290 | 132.8 | 92 | 115.1 | 76.5 | 52.1 | 1 | 1 | 49 | 23.3 | 14.9 | 5.14 | 5.14 |
| 18 | 342 | 140.6 | 91.8 | 124.1 | 79.8 | 49.4 | 2 | 1 | 45.6 | 20 | 15.6 | 9.27 | 4.65 |
|  |  |  |  |  |  |  |  |  | 45.9 | 20 | 16.1 |  |  |
| 20 | 373 | 142.8 | 103.8 | 131.5 | 84.4 | 50.4 | 3 | 2 | 41.1 | 22.7 | 11.8 | 8.95 | 2.98 |
|  |  |  |  |  |  |  |  |  | 42.5 | 21.8 | 11.5 |  |  |
|  |  |  |  |  |  |  |  |  | 38.1 | 21.4 | 10.1 |  |  |
| 11 | 370 | 138.5 | 93.2 | 121.8 | 80 | 52 | ⁄ | ⁄ | ⁄ | ⁄ | ⁄ | ⁄ | ⁄ |
| 12 | 341 | 156 | 95.8 | 123.5 | 82.5 | 51.4 | ⁄ | ⁄ | ⁄ | ⁄ | ⁄ | ⁄ | ⁄ |
| 16 | 299 | 133.1 | 92.8 | 120.1 | 76.7 | 48.7 | ⁄ | ⁄ | ⁄ | ⁄ | ⁄ | ⁄ | ⁄ |
| 19 | 265 | 131.2 | 90.3 | 115.3 | 74.8 | 47.2 | ⁄ | ⁄ | ⁄ | ⁄ | ⁄ | ⁄ | ⁄ |
| Mean±SE | 329.6±11.8 | 136.3±1.5 | 94.4±1.1 | 121.7±1.5 | 78.8±0.9 | 51.3±0.8 | 2.2±0.3 | 1.7±0.2 | 45.5±1.0 | 23.2±0.5 | 14.8±0.8 | 9.47±1.01 | 4.60±0.44 |
