# Supplementary material for: Reproductive ecology of the endangered Beal’s-eyed turtle, Sacalia bealei
Source: PeerJ. 2018 Jun 14;6:e4997. doi: 10.7717/peerj.4997 (PMC6018645; doi:10.7717/peerj.4997)
Supplement: Supplemental Information 3 [file peerj-06-4997-s003.docx]

Table 3 Information on hatchling

| Hatchling ID | Female ID | Egg-laying date | Hatching Date | Incubation Period (Day) | Mean incubation temperature (°C) | Body Weight (g) | Carapace Length (mm) | Carapace Width (mm) | Body Height (mm) | Plastron Length (mm) | Plastron Width (mm) |
| --- | --- | --- | --- | --- | --- | --- | --- | --- | --- | --- | --- |
| 1 | 13 | 19-May | 19-Aug | 92 | 25.17 | 8.0 | 39.0 | 31.9 | 20.3 | 29.9 | 24.9 |
| 2 | 13 | 19-May | 24-Aug | 97 | 25.22 | 8.0 | 37.6 | 25.7 | 15.9 | 29.3 | 22.1 |
| 3 | 14 | 20-May | 22-Aug | 94 | 25.09 | 10.0 | 40.5 | 38.0 | 17.5 | 31.9 | 27.3 |
| 4 | 14 | 20-May | 24-Aug | 96 | 25.11 | 11.0 | 40.1 | 33.5 | 17.4 | 31.3 | 24.0 |
| 5 | 15 | 31-May | 24-Aug | 85 | 25.32 | 10.0 | 39.9 | 35.4 | 17.0 | 32.6 | 27.2 |
| 6 | 15 | 31-May | 30-Aug | 91 | 25.29 | 12.0 | 42.1 | 36.7 | 16.0 | 33.5 | 27.8 |
| 7 | 18 | 03-May | 19-Aug | 108 | 24.33 | 9.0 | 41.5 | 32.1 | 17.5 | 32.6 | 24.3 |
| Mean±SE | | | | 94.7±2.5 | 25.08±0.12 | 9.7±0.5 | 40.1±0.5 | 33.3±1.4 | 17.4±0.5 | 31.6±0.5 | 25.4±0.7 |
